# The sustainable development of Asian students’ project-based learning: Implementing a holistic and indigenous Whare Tapa Rima Model

**DOI:** 10.3389/fpsyg.2022.938931

**Published:** 2022-09-08

**Authors:** Xiudi Zhang, Xiaoming Tian

**Affiliations:** ^1^School of Education Science, Zhoukou Normal University, Zhoukou, China; ^2^School of Humanities and Foreign Languages, Xi’an University of Posts & Telecommunications, Xi’an, China

**Keywords:** Asian students, New Zealand, private training establishment, project-based learning, sustainable development

## Abstract

This study employs a holistic and indigenous theoretical model called Whare Tapa Rima to examine the project-based learning (PBL) experiences of Asian students in a private training establishment, the W institution, at the tertiary level in New Zealand. The analysis shows that Asian students face challenges in their PBL journey in physical, cultural, interconnected emotional and intellectual, social, and spiritual dimensions. Implications from the research analysis may be considered about how to provide better support and international services to Asian students involved in PBL programs worldwide by adopting the responsive, theory-informed framework of the Whare Tapa Rima Model.

## Introduction

New Zealand’s tertiary education sector covers private training establishments, institutes of technology and polytechnics, Wananga (assists the application of Mãori knowledge), universities, and workplace training. In this sector, an increasing number of students are internationals, of whom more than 85% were Asian in origin in 2007 (Campbell and Li, 2008). According to official statistics, international education contributed NZ$4.0 million to GDP from 2015 to 2016 (Education New Zealand commissioned Infometrics and National Research Bureau, 2016). Given the important role that international education plays in the country’s GDP, researching the sustainability of international education in New Zealand is therefore important for local academics, institutions, and government officials to provide better services for sustainable educational development (Zhang, 2020).

The sustainability of international education is often interpreted in several ways in the literature, but two key forms are sustainable education for the institute and that for the student (UNESCO, 2011; Leicht et al., 2018). Sustainable education for the institute often refers to international programs of studies that are going to attract overseas students, provide them with the education they are seeking, and which can ultimately give them useful skills and knowledge that they can utilize after graduation and bring them back to their own countries for application (Hénard et al., 2012). The institute must identify its focus for educational sustainability: what courses and curricula it is going to teach. Many institutes successfully do this, focusing on establishing effective disciplines and well-taught curricula such as English as a Second Language, Food Preparation, Hospitality, or Tourism taught by good academic staff (Lim, 2016; Han, 2021; Kılıç et al., 2021). In this way, an institute establishes a reputation for a good education that international students are keen to receive, thereby sustaining itself as an institute of excellence (Berjozkina and Melanthiou, 2021).

The sustainability of international education also has the student perspective. Naturally, well-taught courses attract overseas students and help the institutes retain them (Mattah et al., 2018). However, students have wider needs than just a good education, extending to learning support, and pastoral support (Spratt et al., 2006; Harris et al., 2020). If an institute has recruited students from abroad, it has a duty to guide them to familiarize themselves with the local area such as where shops are and how to use the public transport, and settle down in the living and learning environment to sustain their overseas study journey (Chavajay and Skowronek, 2008; Ma et al., 2020). For example, the institute can help international students arrange a bank account and associated facilities such as a credit or debit card. It may do this as part of package with its own bank as part of an overall service to the institute (Abdel-Khalek, 2010; Sullivan and Kashubeck-West, 2015; Scotti, 2022). The institute can also offer an orientation that helps international students to familiarize themselves with the school curricula, course system, and learning facilities (Scotti, 2022). In this article, we examine the sustainability of international education from the student perspective. Specifically, we are interested in finding out what hinders the sustainability of Asian students’ project-based learning experience in a private learning establishment of higher education in New Zealand and what support they have obtained to overcome these hindrances along their journey.

We focus on the sustainability of international education in private learning establishments in New Zealand because this topic has been underrepresented. An examination of the relevant literature finds that the majority of the research regarding New Zealand attaches great importance to those international students in universities, rather than in private training establishments (Andrade, 2006; Skyrme, 2007; Abbott and Doucouliagos, 2009). However, private training establishments in New Zealand developed rapidly in the 1980s and 1990s and have been growing in the total number of students in the contemporary period (Education New Zealand commissioned Infometrics and National Research Bureau, 2016). To some extent, private training establishments, as a leading international education industry, play an important role in responding to the need of industries for both international and local graduates with specific skills such as business, hospitality, and travel (Ziguras, 2003).

Therefore, this study chose the W Institution as the research site because it is the first private educational establishment in New Zealand that offers international students with project-based learning (PBL) experience. A wide range of PBL approaches taken by the W Institution includes field trips, visiting speakers, case studies, business plan preparations, and observations. Furthermore, the W Institution encourages learners to take a suitable PBL approach that helps them learn by being involved in real-world and work-integrated projects.

We are particularly interested in the sustainability of Asian students’ project-based learning (PBL) at the W Institution. The PBL approach organizes teaching and learning through projects. More than just thinking about and doing projects; this approach focuses on the process of doing projects that leads to better thinking (Tsybulsky and Muchnik-Rozanov, 2019). In the PBL approach, “projects are curriculum,” providing epistemological and methodological means to direct students toward developing an in-depth understanding of knowledge in disciplines; as such, it calls for active learning from students (Bell, 2010, p. 2). Students engaging in PBL projects need to be active and willing to take the initiative to learn new knowledge by conducting independent research projects (Larmer et al., 2015). Therefore, an active learning approach, rather than passive learning, captures the heart of the PBL approach.

The PBL approach which calls for active learning may not easily be applied to Asian students because these students are often from a sociocultural context that promotes a passive learning style. Specifically, the passive learning style adopted by Asian students who come from countries such as China, Vietnam, Korea, and Japan is inherited from Confucianism (Phuong-Mai et al., 2005). According to Confucianism, listening to teachers has been the most frequent classroom experience of Asian learners (Liu and Littlewood, 1997). Therefore, unavoidable and unexpected problems could exist if Asian learners apply their traditional way of learning in a new context.

Tsybulsky and Muchnik-Rozanov (2019) argue that the idea of assigning projects to Asian students is still a new one even though the PBL approach has been thoughtfully designed and equitably operated as central to the pedagogic paradigm of higher education for nearly 200 years. Shinde and Inamdar suggest that the PBL approach should be adjusted to suit indigenous culture, the history of education, and localized conditions when it is introduced to Asian societies or applied to students with Asian cultural backgrounds (Shinde and Inamdar, 2013). The adjustments include providing support such as proper guidance and supportive infrastructure to students in PBL implementations. This is backed up by Shinde and Kolmos’s (2011) report which suggests the lack of appropriate and necessary guidance has hindered the growth and development of PBL programs in India.

For these international students from Asia, past experiences of schooling shape patterns of learning that do not necessarily fit the New Zealand higher education learning context. Indeed, they need support in a different learning context. In this research, of particular interest is what support they, as learners from Asian cultural contexts, have obtained for sustainable project-based learning in New Zealand. Durie’s model called *Whare Tapa Rima* [WTR] designed to deliver health services to students for sustainable development provides us with the theoretical tools to explore the project-based learning sustainability of Asian students (Durie, 1994). We aim to answer the two research questions: (i) What hinders the sustainability of project-based learning for Asian students in the W institution? and (ii) What support have Asian students obtained from the W Institution to achieve sustainability in their project-based learning?

## Theoretical tool: *Whare Tapa Rima*

**FIGURE 1:**
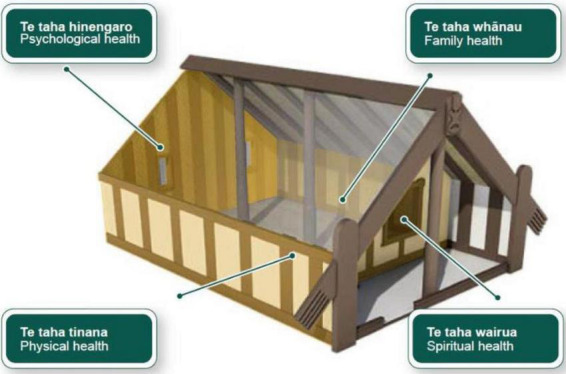
Durie (1994) model of *Whare Tapa Rima*. Reproduced with permission.

*Whare Tapa Rima* [WTR], a model of health service delivery for all-around sustainable support, has been applied to a variety of situations since its birth in 1994 (Durie, 1994). Its application aims to bring in a Mãori cultural perspective to understand the natural world. Underpinned by Mãori philosophy, the model contains four main dimensions: *Taha tinana* (physical dimension), *Taha hinengaro* (the emotional dimension), *Taha whanau* (family dimension), and *Taha wairua* (spiritual dimension; see Figure 1).

Durie’s model has been adapted from the medical field to the educational context with the addition of *Taha Whenua* (the ethnic and/or cultural dimension) and with the updating of *Taha hinengaro* to an interconnected emotional and intellectual dimension (Fielden et al., 2020). This adaptation reflects the recognition of the integrated, relational, and socio-cultural nature of the learning process that characterizes international students’ oversea educational journeys (Fielden et al., 2020). The updated WTR model aims to provide a rich framework that can empower facilitators to value, view, and analyze complex learner PBL needs through inter-related and inseparable physical, cultural, emotional, intellectual, social, and spiritual dimensions and thus provide them with all-around support for sustainable development. The application of the updated WTR model as the theoretical tool enables us to examine the empirical PBL practice in the W Institution and to answer the two foregoing research questions: (i) What hinders the sustainability of project-based learning for Asian students in the W institution? and (ii) What support have Asian students obtained from the W Institution to achieve sustainability in their project-based learning?

## Methodology

Qualitative methodologies are used in this research to offer insights into the meaning that Asian students attach to things in their PBL journeys in the W Institution (Taylor et al., 2015). Qualitative methodologies have been described as naturalistic to some extent (Lincoln and Guba, 1985). This means that researchers adopt strategies that parallel the way in which people act in the course of daily life, typically interacting with informants naturally and unobtrusively (Rallis and Rossman, 2012). Specifically, semi-structured interviewing, as a flexible qualitative technique for small-scale research, has been utilized to collect empirical data (Drever, 1995).

Nine Asian learners (two males and seven females), who studied in the W Institution for at least 1 year, were interviewed. All the interviews were conducted in English and recorded with the participant’s consent, with each interview lasting about 40 min. Interview questions were designed in terms of the two research questions (see above). In addition to this, we also allowed participants to make any complements to the structured interview questions and to share any of their genuine and authentic views and opinions on the topic. We analyze and discuss the empirical findings in the next section.

## Analysis

**FIGURE 2:**
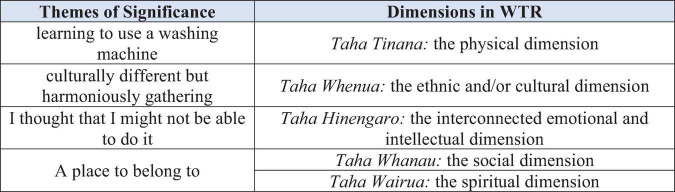
Correspondance between the theoretical dimensions and major empirical themes.

We referred to the five dimensions of the WTR model on which we had drawn to design the semi-structured research questions as a guide for us to code the empirical data of interviews with the nine learner participants. After three rounds of data coding, we categorized four themes of significance; all of the four themes can be framed by the five dimensions of the WTR model, as shown in Figure 2. Next, we report each of the four themes in turn.

### *Taha Tinana:* Learning to use a washing machine

*Taha Tinana* refers to the physical dimension such as “physical state, eating, sleeping, fitness, financial means” (Fielden et al., 2020, p. 10). Our analysis reveals that most of the facilitators in the W Institution attach more importance to providing intellectual, linguistic, cultural, or mental rather than physical support to learners. However, physical support is desperately needed and highly rated by some of the learner interviewees such as Paul and Cathy.

Paul was reported by another student 2 weeks later after he came to the W Institution because he had a strange body smell which was far beyond tolerance. After conversations with Paul, the learners’ manager of the institution found that Paul had not washed his clothes for almost 2 weeks. Paul came from a poverty-stricken remote rural village in Vietnam and had no idea about how to use modern household facilities like a washing machine. The manager solved the problem by offering Paul physical support about how to use the washing machine as well as other facilities in the institution.

Similar to Paul, Cathy also suffered at the beginning of her BPL journey, as she said,

*My project involves gardening and painting skills. In the beginning, I do not know how to do the project due to a lack of physical skills. I am suffering and nearly quit my study. Besides, I am too fearful and dispirited to turn [to] my facilitator for help because I worry that she may think I am so stupid and talentless. Luckily, later when my facilitator notices my problem, she begins to teach me skills by doing such as painting the house and fertilizing flowers. This is indeed a great help to me. I am now greatly confident to continue the project by myself.*

There is not sufficient attention being paid to providing physical support to students even though “physical spaces and other aspects of the physical dimension can promote well-being and learning” (Fielden et al., 2020, p. 10). The lack of physical support resulted in the unexpected PBL learning experiences of Paul and Cathy at the beginning. Both Paul and Cathy are typically passive learners from an Asian cultural context. In Asian culture, especially Confucianism, teachers play a leading role in the class and are expected to be experts who are authoritative in their field of knowledge and convey this knowledge to students (Liu and Chai, 2015). As for learning, students can only ask a question, or make a creation or judgment when they have a solid knowledge foundation (Shen and Shi, 2006; Chen and Sit, 2009). They are expected to learn, recite, and understand a wide body of classics and facts before they can deal with more demanding tasks and use their knowledge freely and creatively (Shen and Shi, 2006). Also, as reflected in a Confucian doctrine, *say yes, when you know; say no when you do not*, students are not encouraged to show off their learning nor pretend to be knowledgeable (Sit and Chen, 2010). Instead, they are expected to read more, recite more, memorize more, think more, and do more, but ask and talk less in class (Shen and Shi, 2006).

When this submissive learning relationship between teacher and learner in the Asian context is negatively transferred to the PBL program within the New Zealand educational context, becoming a tacit norm for Paul and Cathy to deal with their relationships with their facilitators, problems may emerge. For example, Paul worries about asking for physical support. To use his words, “*I am afraid that my facilitator might laugh at me if I ask him about how to use the washing machine.*” Similarly, Cathy is “*too fearful and dispirited scared”* to ask for physical support because she is concerned that her facilitator might think she is too “*stupid and talentless*” to learn new skills.

The issue of physical adjustment and adaptation is always a pressing concern for international students in a non-native cultural context (Poelzl, 2012). The W Institution’s failure or inability to support Paul and Cathy physically at the beginning has put both students under academic and living pressures. The UNESCO calls for incorporating the principles, values, and practices of sustainable development into all aspects of education and learning (UNESCO, 2005). Physical support, as one learning aspect, was easily ignored by the education provider. Fortunately, the physical support of the learners’ manager to Paul, and the project facilitator to Cathy has helped to ameliorate their difficulties so that they can continue their PBL journey at the W Institution.

### *Taha Whenua: Culturally* different but harmoniously gathering

*Taha Whenua* denotes the ethnic/cultural dimension, referring to the genetic and environmentally created cultural way in which we approach our life (Fielden et al., 2020, p. 10). New Zealand has an ethnically diverse population. According to the country’s most recent national census in 2018, 74% of the population was identified as European and 16.5% as Mãori. Other major ethnic groups include Asians (15.1%) and Pacific peoples (8.1%; Stats NZ, 2018). The statistics also cover overseas students in local educational institutions at different levels.

In such a culturally pluralistic society, international students, to some extent, inevitably come across cultural sensitivity. Therefore, the provision of culturally friendly space for students not only supports their need for understanding cultural diversity but also helps to minimize their alienation from *different worlds* of school culture (Heath, 1983; Ladson-Billings, 2009). This is seen in our interview with Catherine.

*I remembered our school held a cultural event in the first year I came here. I clearly remembered lots of students attended the event no matter where they come from, like India, Sri Lanka, the Philippines, and New Zealand. The event shows us how different cultures, especially my own culture, are respected by the school, and how we are culturally different but still, we can work together and stay together harmoniously. So, it was indeed a good event. I felt happy that the school held the event because it allows me to know people around me and make friends with them. Some of us become close and intimate friends now and they contribute to my project.*

Little argues that schools could become involved with cultural diversity by, for example, taking a multicultural approach to community involvement which allows students and teachers to seek ways to become involved in the school cultural environment (Little, 1999). The W Institution holds a cultural event for students, seeking participatory, active, and engaging interactions and involvement from the students. The event provides a platform for students from different cultural worlds to get to know each other. In this research, Catherine revealed that she had made some friends through the school cultural event and some of these friends later contributed to her project. What is more important to Catherine is that the event showed how the W Institution respects each culture, in her words, *“how different cultures, especially my own culture, are respected by the school.”* Such a cultural event helps to create a harmoniously multicultural school environment and thus avoid “cultural blindness,” a term used to categorize “any policy, practice, or behavior that ignores existing cultural differences or that considers such differences inconsequential” (Robins et al., 2006, p. 89). Cultural blindness may likely deprive minority learners of their possible success in school or cause undesired harm to them by imposing on them the sense that their culture is not visible (Howard-Hamilton, 2000; Robins et al., 2006; Nieto, 2007).

The W Institution’s culturally responsive administration is also seen in how it provides learning projects about cultural studies, as seen in our interview with Jean.

*My project is about multicultural research. One thing that I learned from the project is how to manage and respect different cultures and ethnic beliefs. Most of the learners that I encountered in the project are internationals from different cultural and ethnic backgrounds. In the process of getting along with them, I learned how to treat different cultures equally and how to get along with people from other parts of the world with a positive and respectful attitude. I have not realized the importance of respecting cultural diversity before doing the project.*

Jean attests to the fact that her project enables her to notice the importance of understanding and respecting cultural diversity, which is something important but was neglected by her before the project. This result may be far beyond the wildest dreams of the project designer of the W Institution. Gollnick and Chinn (2009) suggest that 21st-century classrooms require a new type of teaching and learning which considers multicultural and multilingual characteristics. These characteristics not only refer to traditions, languages, value ideals, and cultural beliefs but also extend to the communitive skills, cultural competencies, and learning styles of students who are culturally and ethnically diverse (Gay, 2002).

The W Institution inserts the multicultural dimension into some of its projects as part of the curriculum content and uses them as “conduits” to teach learners more effectively (Gay, 2002, p. 106). These cultural projects provide a learner with opportunities to break any stereotyped or biased understandings toward other cultures through re-examining his/her deeply held ideals, values, and beliefs about his/her own culture and other cultures as well, especially those non-mainstream ones (Villegas and Lucas, 2002; Grant and Asimeng-Boahene, 2006; Nieto, 2007). In our research, Jean’s project helps her to realize the importance of respecting cultural diversity.

Furthermore, the W Institution’s cultural support in both forms of academic projects and cultural events helps to equip learners with practical experience in performing career-specific skills and tasks; this increases their confidence and a sense of responsibility and accountability in the multicultural work environment (Jackson, 2016). For instance, in our after-interview correspondence with Jean, she suggests that the intercultural competence developed from the PBL experience has contributed to her confidence and capabilities in the workplace. To use her words, “the school project teaches me strategies to get along well with and respect my colleagues from diverse cultural contexts.” This finding resonates with an American official social report which suggests that the lack of intercultural competencies may result in social and civic consequences like underemployment, limited civic participation, and health risks (Schott Foundation for Public Education, 2009). Therefore, to facilitate student learning, educators require a deep understanding of students’ existing sustainability dispositions that influence their ability and willingness to develop the required competencies and agency to contribute to sustainability transitions.

### *Taha Hinengaro*: I thought that i might not be able to do it

*Taha Hinengaro* denotes the interdependent intellectual and emotional dimension, including “thinking and feeling capabilities, responses and nature” (Fielden et al., 2020, p. 10). Our analysis suggests that the emotion/mental state and the intellectual aspect influence each other, affecting some of the interviewees’ PBL experience in the W Institution. This impact is made visible in our interviews with Jenny and Seema. Jenny came to New Zealand for a brighter future after obtaining her master’s degree from a Vietnamese university. She encountered some mental problems at the outset of her PBL journey in the W Institution. We provide a long quotation of data here because Jenny’s words are worthwhile for deeper analysis.

*I was extremely nervous at the very beginning of the project because I knew nothing about it. I had no idea at that moment whether I could overcome the anxiety and become a qualified student. I even began to suspect that my existing knowledge foundation was not solid at all for the use of the project because doing research here is quite different from my past research experience in Vietnam. I had completed a research project in Vietnam for my master thesis. In that project, I did not need to go to fieldwork, interview, and collect data. All that I needed was to write up the thesis. We call it a research project. Fortunately, when I communicated my anxiety with my facilitator, her words eased me. She asked me to relax and not to be nervous. She told me that I was the only master student she had taken over the years and my past academic experience would definitely be useful for the project. Her words helped me re-recognize the value of my past academic experience, eased my anxiety about the unknown project, and regained confidence in the project. Later when I was about to end the project, I realized that my facilitator was correct. I was abnormally nervous when I first started the project.*

An interesting finding is that Jenny felt “*extremely nervous*” at the beginning of her project even though she had already obtained a master’s degree before she arrived at the W Institution. Confronted with an unfamiliar project, Jenny became unconfident and even began to doubt herself and her knowledge base. This indicates that Jenny’s emotional state is closely related to her identification and recognition of her academic competence. Moreover, this unfamiliar academic environment, different learning styles, and discrepant definitions of research also aggravated her mental anxiety and academic insecurity; thus, she to some extent lost her self-identity at the beginning of her PBL journey at the W Institution.

Giddens (1991) described self-identify as having a rational understanding of what doing is and of the rationale behind doing. This implies the requirement for reflexive consciousness. The PBL approach is argued to be an ideal way to develop the learner’s self-identity given that a project can stimulate and initiate learners’ self-identity by making sense of experiences, practice, and work (Giddens, 1991). However, this approach may also make one lose self-identity even before a project starts if one has strong mental anxiety toward the unknown project and is not assisted in a timely manner, as shown in Jenny’s case. Under such conditions, external support is particularly important. In this study, Jenny’s facilitator helped her alleviate her anxiety about the unfamiliar project and return to a rational understanding of her academic self-identity. In addition to mental help, our analysis suggests that writing a reflective diary might be an effective method to understand self-identity rationally, as is shown in the interview with Seema.

*My facilitator asked me to keep a reflective diary as a way to understand myself and my project. I was very reluctant to do so at the beginning. But now I think writing a reflective diary is very conceived to carry out the project. Because when I reflect on my project work every day, I not only focus on what I have achieved but also consider things like the reasons why I did not do some project work well, how I should improve in the next step, and what I can do further to challenge myself. For example, I often imagine how I would behave if I were put in an unknown situation or an unknown environment to solve an unknown problem or cope with an unknown difficulty in my diary.*

Seema’s case reveals that keeping a reflective journal has a positive impact on learners to gain a clear self-awareness, to make academic progress through projects, and to prepare for unknown situations. Eraut argues that non-conscious learning and tacit knowledge need to be made explicit through collective reflective dialogs so as to share practice knowledge and develop expertise (Eraut, 2000). In this research, reflections on emotional and intellectual aspects have led to Seema’s satisfaction with herself and her project. Both Jenny and Seema have received support from their respective facilitators, which helps them continue their respective projects smoothly. Our analysis of the two cases indicates that self-identity develops with and is shaped by learners’ experiences. In this process, support from outsiders may contribute to this development.

### *Taha Whanau* and *Taha Wairua:* A place to belong to

*Taha Wairua* denotes the spiritual dimension, referring to “beliefs, values and attitudes or spiritual, religious and/or moral commitments that students have” (Fielden et al., 2020, p.11). Our investigation suggests that the two dimensions are connected in terms of providing a sense of social/spiritual belonging to the participants, as seen in our interviews with Sally, Isaac, and Judy.

*Sally: I changed my major from tourist studies to mathematics teaching. This new major calls for more professional knowledge. Therefore, I faced huge academic challenges at the beginning. I could only turn to my facilitator for help because I only knew him at the beginning. Luckily, the school later supported me to visit Te Kura to explore mathematics teaching strategies in a real New Zealand pedagogic context. This allows me to make friends with some mathematics teachers. I also exchanged lots of ideas on mathematics teaching with them. All of these give me a sense of academic belonging.*

International students have less opportunity to establish social support networks among peers and are more likely to rely on their academic programs, especially faculty (Mallinckrodt and Leong, 1992; Sullivan and Kashubeck-West, 2015). This is seen in Sally’s case that she “*could only turn to [her] facilitator for help because [she] only knew him at the beginning.”* Under such conditions, social support from the W Institution has helped to solve the problem and provided Sally with “*a sense of academic belonging.*” In addition to social support, we find that spiritual support also contributes much to the well-being of Asian learners, as is demonstrated by our interviews with Isaac and Judy.

Isaac is from India and has a strong belief in Hinduism. At the beginning of his PBL journey, he was struggling to find a *spiritual home* where he would belong in New Zealand. Noticing this, his facilitator helped him join a Hinduism group in the vicinity of the W Institution. Isaac highly rated this in the interview, thinking that his facilitator has helped him find a *new home* to inhabit in New Zealand.

*Isaac: whenever I encounter difficulties in the operation of the project, I can always come to my new home to talk about my difficulties. I can always gain encouragement and comfort from the home members. They can always help me to regain my courage and confidence to continue my project.*

The support from Isaac’s facilitator is effective in that it not only gives Isaac a sense of spiritual belonging but also helps to alleviate his academic burdens and to regain courage and confidence to continue the project. Such support regarding the spiritual dimension also proves helpful to Judy. Judy is from the Philippines. She regards New Zealand as a place to escape from what she described as *trauma –* her husband had just divorced her before she left the Philippines,

*The project itself was too difficult and required a lot of time and energy and I had no way of starting it at the beginning. What was worse, I had no time to care about these academic difficulties because I was so sad, so sad! And sometimes I felt that I could not even breathe. Then my facilitator came to me, comforted me, and recommended me to join a Christian church, which gradually relieved my trauma. I can now live a normal life.*

In Judy’s case, the spiritual support from her facilitator has helped to heal her trauma and for her to live a sustainable normal life. The three cases of Sally, Isaac, and Judy demonstrate that incorporating the dimension of social and spiritual well-being in students’ lives to enhance a sense of peace, hope, faith, and comfort can lead to increased levels of happiness and life satisfaction, which, in turn, helps to contribute to their PBL journeys in the W Institution.

As argued by Ortega-Sánchez and Gómez-Trigueros, desired outcomes and solutions to existing challenges to sustainability cannot be produced if the awareness of problems does not reflect the inner needs of individuals and does not support by appropriate education (Ortega-Sánchez and Gómez-Trigueros, 2019). This also resonates with existing research findings that social/spiritual support often leads to positive effects such as better learner adjustment (Kneipp et al., 2009), stronger engagement in health-promoting behaviors (Hsiao et al., 2010), a better quality of life, and higher levels of happiness and social well-being (Abdel-Khalek, 2010). Ultimately, this is the goal of sustainable development that ensures the success of students in their future careers by providing them with the skills, motivations, and a set of values that enable them to contribute to the well-being of the global community. Therefore, higher education institutions are centers of support and value transfer at which future generations will build a sustainable world.

## Conclusive thoughts

This research is part of a wider project of us to promote Sustainable Development Goals in Higher Education Institutions. The study reveals different hindrances (physical, cultural, emotional, intellectual, social, and spiritual) that prevent Asian learners from sustainable development in their PBL journey in New Zealand, although the PBL approach has been widely taken as an effective pedagogic method in higher education globally. This resonates with Goodson and Lindblad (2011) who argue that the PBL curriculum is implemented and understood unconsciously in quite different ways in different sociocultural contexts.

As a case study, the W Institution has provided holistic support, including physical, cultural, emotional, intellectual, social, and spiritual dimensions to the interviewees, in various forms and to a different extent. These different types of support have proved effective in helping our participants to overcome the hindrances, thereby maintaining the sustainability of their PBL journey and ensuring that sustainable development becomes an integral part of general education (Gomes et al., 2022). Our analysis also implies the usefulness and effectiveness of the WTR model in delivering holistic ingenious and indigenous support and resources to international students that improves their well-being and project-based learning sustainability in the New Zealand context.

There might be a reserved research limitation that the research sample of this study came from only the W institution. This means that the results cannot be generalized to all international students at private learning establishments in New Zealand. It is important, however, that the analysis took into account the dependencies on the field of study studied. Moreover, non-probabilistic selection methods based on a convenient selection of the sample were used, which has an impact on drawing general conclusions. The resulting limitations could be reduced by inquiring further into this research topic.

## Data availability statement

The original contributions presented in this study are included in the article/supplementary material, further inquiries can be directed to the corresponding author.

## Ethics statement

The studies involving human participants were reviewed and approved by the Ethics Committee of School of Education Science, Zhoukou Normal University, Zhoukou, China. The patients/participants provided their written informed consent to participate in this study.

## Author contributions

XZ contributed to the conception, design of the study, organized the database, performed the statistical analysis, and wrote the first draft of the manuscript. XT validated the statistical analysis, wrote and revised sections of the manuscript, contributed to the manuscript revision, read, and approved the submitted version. Both authors contributed to the article and approved the submitted version.
